# Impedance-Matched Iron-Added Polymeric Composite Film Incorporated with Iron Nanowire for Electromagnetic Absorption Application

**DOI:** 10.3390/polym17212965

**Published:** 2025-11-06

**Authors:** Yuh-Jing Chiou, Pei-Jung Chang, Pei-Ru Su, Sheng-Jung Tsou, Chung-Kwei Lin

**Affiliations:** 1 Department of Chemical Engineering and Biotechnology, Tatung University, Taipei 104, Taiwan; chiou@gm.ttu.edu.tw (Y.-J.C.); peronchang@tmu.edu.tw (P.-J.C.);; 2 Research Center of Digital Oral Science and Technology, College of Oral Medicine, Taipei Medical University, Taipei 110, Taiwan; 3 Graduate Institute of Manufacturing Technology, National Taipei University of Technology, Taipei 106, Taiwan; 4 School of Dental Technology, College of Oral Medicine, Taipei Medical University, Taipei 110, Taiwan

**Keywords:** impedance matching, sheet resistance, iron powder, polymeric composite, sheet resistance adjustment

## Abstract

Salisbury screen-type radar absorption structures (RASs) consisting of a resistance sheet, a spacer, and a conductive base provide an efficient method for microwave absorption. An impedance-matched resistance sheet allows microwaves to enter, whereas superior microwave absorbers enhance their performance further. In the present work, an impedance matching composite film was prepared by using polymer/iron/iron nanowires. By varying the polymer, poly (methyl methacrylate) (PMMA), poly (vinylidene fluoride) (PVDF), and poly (vinyl alcohol) (PVA), to iron powder ratios (1:1, 2:1, and 4:1), composite films were synthesized and examined by scanning electron microscopy, X-ray diffraction, and the four-point probe method to determine the materials’ characteristics. An impedance-matched composite film was prepared based on the selected composition with 1–10 wt.% iron nanowire additions. Experimental results showed that the polymeric composite film prepared by a ratio of iron-PVA of 4:1 exhibited a sheet resistance of 49 ± 9.7 Ω/sq due to well dispersion of iron powder in PVA. With 1 wt.% Fe nanowire addition, the optimal composite sheet resistance was 329.7 ± 45.3 Ω/sq, which corresponded to an impedance matching degree (i.e., |Z_in_/Z_0_| value) of 0.88 ± 0.12 and can be used as a resistance sheet for a Salisbury screen-type absorber in RAS applications.

## 1. Introduction

Electromagnetic interference has become an important issue and is highly emphasized in modern society. Whether in civilian or military applications, appropriate absorption is necessary to prevent harmful effects such as harm to human health, signal interference, damage to equipment, or being detected by military [1,2,3,4]. In the military application, absorbing the microwave is the main strategy that can improve the survivability of aircraft and ships [5,6]. Lots of technologies have been reported, including plasma stealth [7], stealth structure design [8,9], stealth pattern design [10], etc., for efficient microwave absorption.

In the application of radar stealth technologies, radar absorption materials (RAMs) and radar absorption structures (RASs) play important roles in efficiently absorbing electromagnetic wave energy [11,12,13,14,15,16] to reduce the possibility of detection [17]. RAMs absorb microwaves based on their electromagnetic properties, whereas RASs minimize reflection through structural design and impedance matching. The combination of materials and structural properties manipulates the overall absorption performance. Among them, RASs have been widely researched and developed due to their superior mechanical and microwave absorption properties [18]. For instance, foam structure [19,20], honeycomb structure [21,22,23], pyramid structure [3], and Salisbury structure [24,25] have been investigated. In the RASs of Salisbury screens, a resistance sheet is placed in front of a conductive base with a spacer between them [26]. With the air-impedance matching of the resistance sheet (~377 Ω/sq), perfect transmission of microwave energy is achieved [27]. The transmitted wave energy further passes through the air spacer and is reflected by the conductive base. According to the phase shift, the reflected wave energy cancels with the transmitted wave energy, resulting in effective absorption [28].

All RASs must possess the impedance matching property that allows microwaves to enter [16,29,30,31,32,33,34]. With an excellent microwave absorber within the RASs, microwave absorption performance can be largely improved. Even if the incident wave passes through the resistance sheet, the spacer thickness (equaling λ/4 of incident wave) limits the operation for radar absorption [35,36]. According to the reported research, Liu et al., through stacking reduced graphene oxide sheets to form the Salisbury screen-type absorber, studied the relationship between stacking thickness and microwave absorption performance [37]. The finding showed that the high stacking thickness exhibited a larger dielectric constant, resulting in the best reflection loss within a thin spacer thickness of 1.9 mm. Chen et al. prepared different CNT contents of large-area CNT composite papers as an impedance-matching resistance sheet. The optimal 5 wt.% CNT composite paper exhibited a minimum reflection loss of −16 dB at 7.5 GHz with a spacer thickness of 3.0 mm [38]. Recently, Tsyhanok et al. reported the investigation of carbon–carbonyl iron composites prepared by 3D printing [39]. According to the calculation of the absorption performance in a Salisbury screen-type absorber, the reflection loss was less than −33 dB with an addition of 46 wt.% carbonyl iron and a thickness of 0.256 mm. An impedance-matched resistance sheet with excellent microwave absorber additions, however, was seldom investigated.

Numerous studies have focused on developing efficient absorbing additions to enhance microwave attenuation [40,41]. In this context, polymers have been widely used as matrix materials in microwave absorbers, due to their light weight, flexibility, and adjustable dielectric properties [42,43]. For instances, Pavlou et al. used poly (methyl methacrylate) (PMMA) as a polymeric matrix substrate to deposit centimeter-sized graphene. The so-obtained graphene-polymer nanolaminates exhibited high electromagnetic shielding effectiveness values in the terahertz frequency region [44]. Im et al. mixed graphene nanoplatelets, Ni, and PMMA to develop a multilayered graded-structured composite [45]. Reflection was effectively minimized in the multilayered graded structure by controlling the composition of graphene nanosheets and Ni in the PMMA matrix to adjust the conductivity of the first layer. The shielding efficiency approached ~61 dB in the X-band region. Similar designs of multilayered graded structures have been explored by Yu et al. who prepared poly (vinyl alcohol) (PVA)/carbon aerogel composites [46]. Superior absorption performance was achieved by allowing the microwave to enter the absorber through tuning the electromagnetic properties of each layer. Zhao et al. used poly (vinylidene fluoride) (PVDF) as a polymeric matrix to prepare PVDF/multiwalled carbo nanotube nanocomposite foam [47]. Through designing the void fraction of the nanocomposite foams, conductivity and dielectric permittivity were adjusted. A minimal refection loss of −34 dB was achieved with a thickness of 1.7 mm. Furthermore, various studies have addressed the different polymeric matrices on microwave absorbers, such as epoxy, paraffin, PVA, PVDF, and so on [2,46,48,49,50]. However, investigations specifically addressing the role of material composition in Salisbury screen-type absorbers remain insufficiently explored, reflecting current challenges in understanding and optimizing resistance sheet materials in impedance matching.

In research on RASs, the Salisbury screen-type absorbers have long been recognized as a classical structure where the absorption performance depends on the impedance matching among the sheet resistance, permittivity, and permeability [51,52]. In aerospace engineering-related studies of the Salisbury screen-type absorber, resistance sheets are typically evaluated using the free-space measurement method to assess the overall absorption performance [27]. However, investigations focusing on the modulation of sheet resistance and its direct effect on impedance matching are still limited. Similarly to those reported in the literature, our previous studies were mainly focused on the performance RAMs [53,54,55]. With a focus on adjusting the sheet resistance rather than analyzing permittivity or permeability behaviors, this study was the first attempt try to integrate the absorbing materials into the design of an impedance-matched resistance sheet. In the present work, the commercial iron powder was added to three different polymer matrices, PMMA, PVDF, and PVA to adjust the sheet resistance of the composite film by varying the amount of iron powder. Fe nanowire microwave absorbers [55] were further added to manipulate polymeric composite film to approach impedance matching (~377 Ω) for its potential Salisbury screen-type RASs application. This approach provides a novel design strategy and offers new insights into the development of impedance-matched polymeric composite film from a sheet resistance-control perspective.

## 2. Materials and Methods

### 2.1. Preparation of Iron-Added Polymeric Composite Film

The poly (methyl methacrylate) (PMMA, M_w_ ~ 15,000, Sigma-Aldrich Co., Burlington, MA, USA), poly (vinylidene fluoride) (PVDF, M_w_ ~ 180,000, Sigma-Aldrich Co., Burlington, MA, USA), and poly (vinyl alcohol) (PVA, M_w_ ~ 98,000, Sigma-Aldrich Co., Burlington, MA, USA) were separately used as polymer matrices that were dissolved in suitable solutions. The PMMA, PVDF, and PVA were separately dissolved in dichloromethane (DCM, Toronto Research Chemicals Inc., Vaughan, VA, Canada), N-methyl pyrrolidinone (NMP, Seedchem, Box hill, Victoria, Australia), and deionized water, respectively. Afterwards, a commercially available iron powder (Shimakyu Co., Ltd., Niigata, Japan) was used as additives for adjusting sheet resistance. The polymer matrix with different weight ratios of iron to polymer (1:1, 2:1, and 4:1) were chosen to explore the effect of iron powder content on the sheet resistance properties to reveal a suitable composition range for impedance-matching optimization. The selected ratios were mixed by mechanical stirring with a constant rate of 800 rpm for an hour.

**Figure 1:**
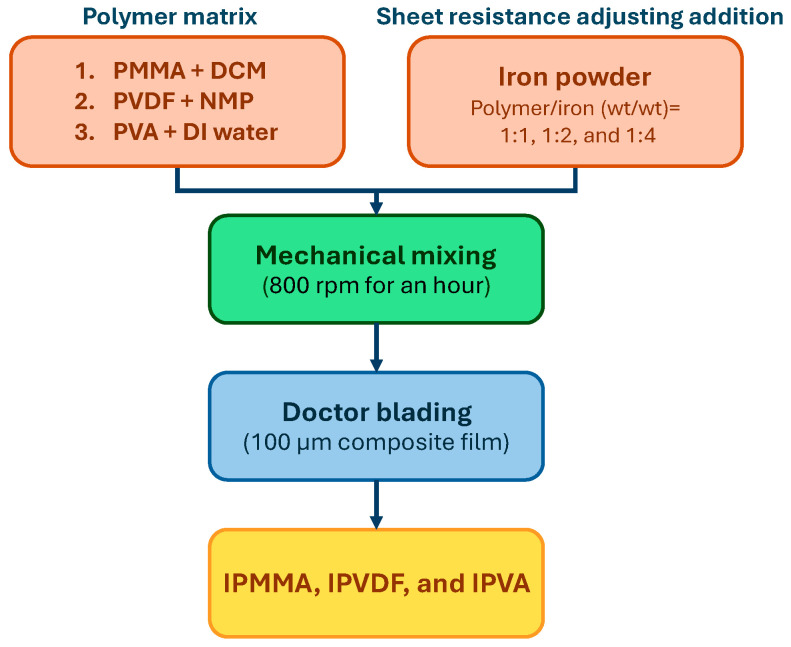
The experimental flowchart for the preparation of the polymeric composite films.

The so-obtained composite slurries were placed on a flat surface and a 100 μm doctor blade was used to prepare the composite film with a dimension of ~20 × 20 cm^2^. Thereafter, the composite film was dried in an ambient environment within a chemical hood. The thicknesses of all prepared composite films were verified using a film thickness gauge. Due to the slight shrinkage by using the doctor blading method, a thickness deviation within 5% was considered acceptable. The so-obtained iron-added polymeric composite films were coded as Iron-PMMA, Iron-PVDF, and Iron-PVA, respectively. Figure 1 shows the preparation flowchart of the polymeric composite films.

### 2.2. Synthesis of Fe Nanowires

Fe nanowires were used as microwave absorbers to adjust the sheet resistance of the selected iron-added polymeric composite films. Fe nanowires were synthesized according to the previous report [55]. A 1M FeCl_2_·6H_2_O (Sigma-Aldrich Co., Burlington, MA, USA) solution was placed into a parallel magnetic field by a glass reactor (11.5 × 4.5 × 30.0 cm^3^). The magnetic field, which was >2000 Gauss, was obtained by two NdFeB magnets (15.0 × 15.0 × 2.5 cm^3^) placed apart with a distance of 5.0 cm. Before adding the reducing agent, the solution was purged with a mixed gas of hydrogen/argon to remove the oxygen from the reactor for 30 min. The 1M NaBH_4_ (First Chemical Works, Taipei, Taiwan) reducing agent solution was slowly added to the Fe ion-containing solution with a continuous hydrogen/argon mixed gas inlet. After finishing the addition of the NaBH_4_ solution, the reactor was kept purging for 5 min to make the Fe ion reduction reaction complete. The Fe nanowires were removed from the solution and washed with a citric acid-containing acetone (1 g in 500 mL) with a magnet that was placed outside the reactor. After washing several times, the Fe nanowires were placed in a tube furnace and heat-treated at a temperature of 250 °C for 1.5 h under a mixed gas atmosphere of hydrogen and argon, and then collected.

### 2.3. Characteristics of Iron-Added Polymeric Composite Film

The abovementioned materials (including iron powder, PMMA, PVDF, and PVA) were examined by X-ray diffraction (XRD), scanning electron microscopy (SEM), and Fourier transform infrared spectroscopy (FTIR). The crystalline structure of iron powders and iron-added composite films were examined by an X-ray diffractometer (D2 phaser, Bruker, Billerica, MA, USA). The operation parameters were set at 40 kV and 30 mA with a nickel-filtered CuKα radiation and the XRD patterns were recorded from 2θ = 15° to 85°. The powder and surface morphologies were observed by a cold-emission SEM (SU 8000 Series UHR, Hitachi, Marunouchi, Chiyoda-ku, Tokyo, Japan). The surface morphologies of the iron-added composite films were observed by an optical microscope (BX60M, Olympus Corporation, Hachioji, Tokyo, Japan). The functional groups of polymer matrices were examined by FTIR (Spectrum GX, PerkinElmer, Inc., Shelton, CT, USA). The FTIR sample was prepared by dispersing polymer powder (1 wt.%) in dry KBr (Fisher Scientific, Waltham, MA, USA). The testing samples were pressed with a pressure of ~ 10 Torr using a circle mold (ø = 13.0 mm). The FTIR spectra were recorded within a range from 4000 cm^−1^ to 400 cm^−1^. Furthermore, the inVia™ confocal Raman spectroscopy (Renishaw, Wotton-under-Edge, UK) was used to determine the bonding of the iron powder. The 514 nm He-Ne laser was used as an excitation source with a power of 9.1 mW. The sheet resistances of composite films were measured by a four-point probe (KeithLink Technology Co., Ltd., New Taipei City, Taiwan) with a rectangular shape (4.8 × 9.6 cm^2^) and a thickness of 100 μm. For each sample, measurements were performed at three different positions, with each position separated by at least 1 cm to ensure spatial uniformity. Additionally, five replicate specimens of the same composition were tested to account for variability between samples. The final reported values were the averages of all measurements with standard deviations to indicate the experimental uncertainty.

## 3. Results and Discussion

### 3.1. Characterization of Iron Powder-Added Polymeric Composite Films

**Figure 2:**
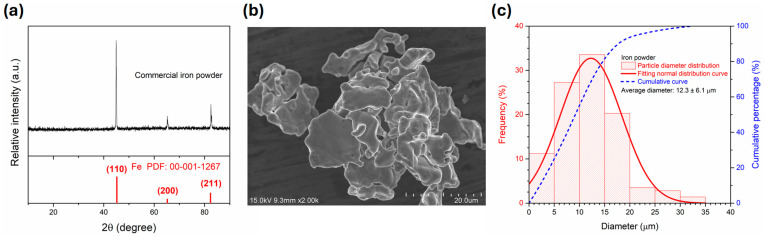
(**a**) X-ray diffraction curve, (**b**) SEM image, and (**c**) particle size distribution of commercial iron powder.

Figure 2 shows the characteristics of the iron powder. The X-ray diffraction pattern of iron powder was illustrated in Figure 2a where the characteristic diffraction peaks of α-Fe (body-centered cubic, ICDD PDF No. 00-001-1267) were observed at 45.2°, 65.2°, and 82.4° for the crystalline planes of (110), (200), and (211), respectively. The average crystalline size of iron powder was 64.2 ± 18.9 nm and was calculated using the Scherrer’s formula [56]. Figure 2b shows the SEM image of iron powder that exhibited irregular flake-like shape with obvious agglomeration. The particle size distribution was further measured by Image J and the result was shown in Figure 2c. The average particle size of iron powder was 12.3 ± 6.1 μm.

**Figure 3:**
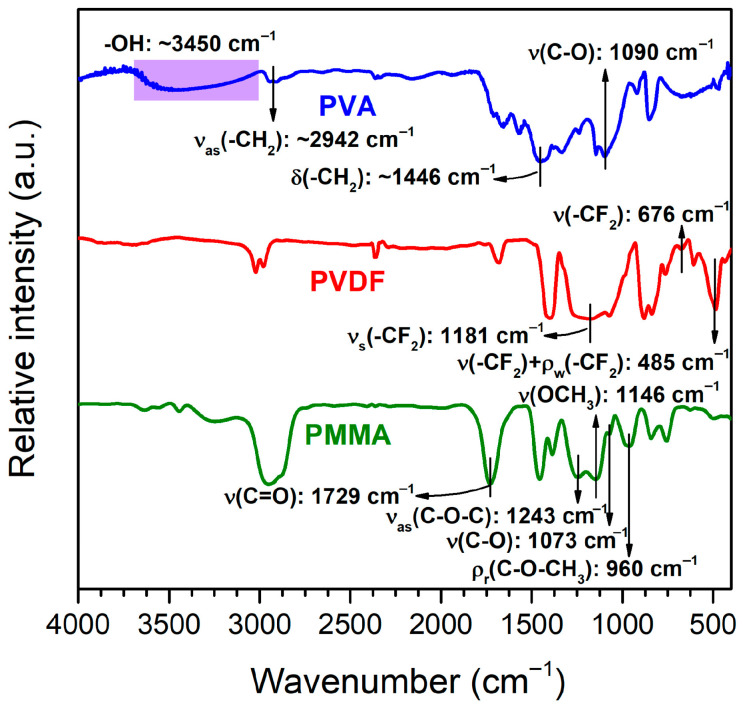
FTIR patterns of a commercial PMMA, PVDF, and PVA matrix.

Figure 3 shows the FTIR patterns of PMMA, PVDF, and PVA. From the PMMA (green line), the characteristic absorbing bands were observed at 960, 1073, 1146, 1243, and 1729 cm^−1^ for ρ_r_(C–O–CH_3_) (rocking), ν(C–O) (stretching), ν(OCH_3_) (stretching), ν_as_(C–O–C) (asymmetric stretching), and ν(C=O) (stretching), respectively. This shows a similar result as reported by Wu et al. [57]. The red line in Figure 3 shows the FTIR pattern of PVDF. The characteristic bands 485, 676, and 1181 cm^−1^ correspond to mixed vibrational modes of stretching (ν) and wagging (ρ_w_) on –CF_2_, a single mode of v(–CF_2_), and a symmetric stretching mode (ν_s_) of –CF_2_, respectively [58]. The last polymer matrix, PVA (blue line), exhibited a broad -OH functional group at ~3450 cm^−1^ and three specific absorbing bands at 1090, ~1446, and ~2942 cm^−1^ that can be attributed to ν(C–O), δ(–CH_2_), and ν_as_(–CH_2_), respectively. This shows a typical PVA FTIR spectrum [59].

**Figure 4:**
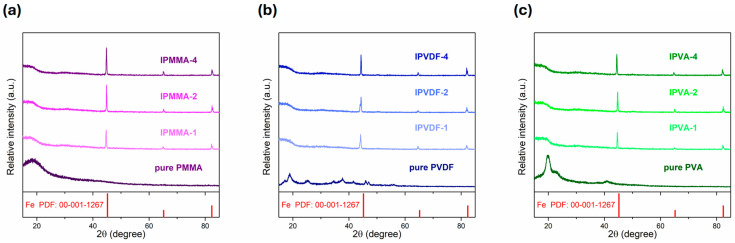
The X-ray diffraction patterns of (**a**) PMMA, (**b**) PVDF, and (**c**) PVA with various iron powder added ratios (pure, 1:1, 2:1, and 4:1).

The polymer matrices were mixed with iron powder in various weight ratios (1:1, 2:1, and 4:1) and formed a series of composite films. The crystalline structure of these films were examined by XRD. Figure 4a shows the XRD patterns of PMMA with various amount of iron additions. It can be noted that pure PMMA exhibited a broad diffraction peak centered at ~19°. According to the abundance of functional groups of methyl (–CH_3_) and ester methyl (CH_3_–O–CO–) at the chain side, the PMMA chains were hard to pack together and exhibited an amorphous structure [57,60]. After adding iron powders, the Fe characteristic peaks were observed and the intensities increased with the increasing amount of iron addition. As shown in Figure 4b, the XRD patterns of pure PVDF exhibit two relatively sharp diffraction peaks at 18.6° and 20.2°, and two broad peaks at ~26.6° and ~39.1°, respectively. These diffraction peaks represented the semi-crystalline characteristics of the α-phase (at 2θ = 18.6°, 26.6°, and 39.1°) and β-phase (at 2θ = 20.2°) in PVDF [61,62,63]. Similar XRD results were reported by Morali et al. [64]. After mixing with iron powder, the crystalline peaks of PVDF became ambiguous. This suggests that the structure of PVDF may be affected by the iron powder. For pure PVA film, as shown in Figure 4c, diffraction peaks were observed, respectively, at 20.1°, 23.0°, and 40.6° for the crystalline plane of (101), (200), and a mixed diffraction peak from planes of (110), (111), and (211) [65,66]. A similar trend as those of Iron-PMMA and Iron-PVDF was observed for various Iron-PVA. It is interesting to note that, as shown in Figure 4, after adding iron powder similar XRD patterns were observed for polymeric composite films.

**Figure 5:**
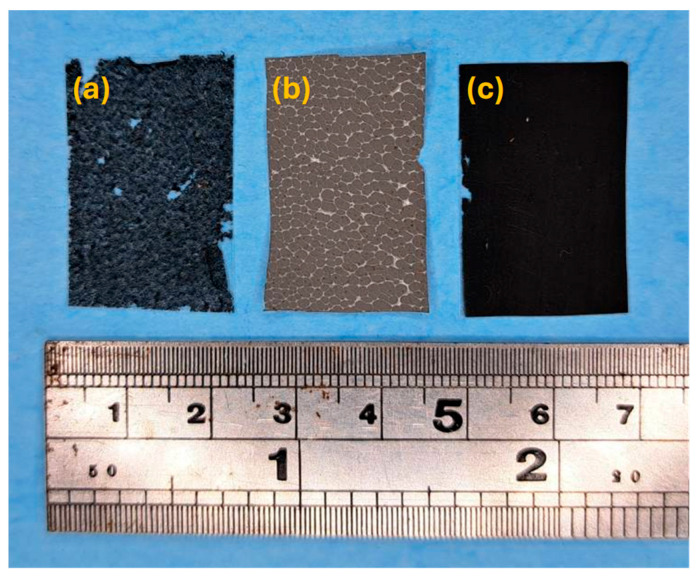
The photos of (**a**) IPMMA-4, (**b**) IPVDF-4, and (**c**) IPVA-4 composite films.

**Figure 6:**
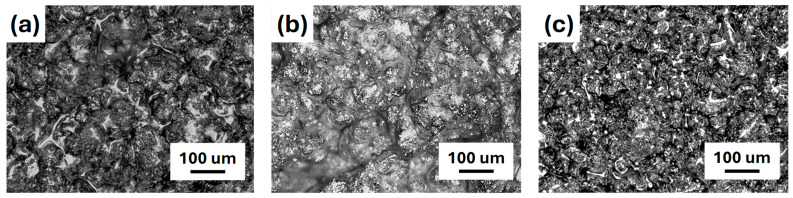
OM images of (**a**) IPMMA-4, (**b**) IPVDF-4, and (**c**) IPVA-4 composite films.

**Figure 7:**
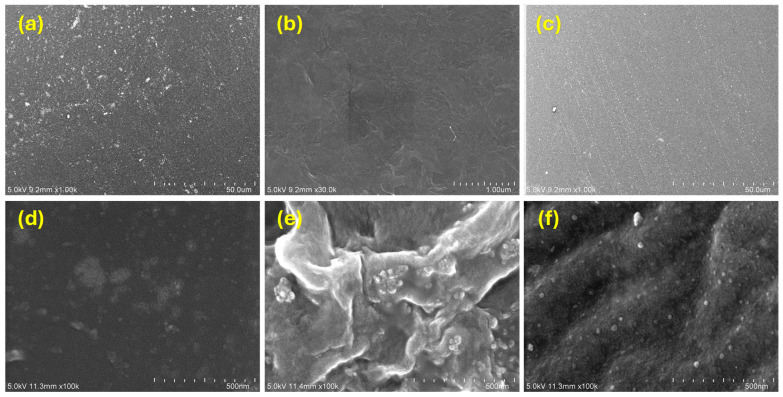
SEM images of (**a**,**d**) IPMMA-4, (**b**,**e**) IPVDF-4, and (**c**,**f**) IPVA-4 composite films with low (**a**–**c**) and high (**d**–**f**) magnifications.

Iron-added composite films prepared with a weight ratio of 4:1 were first examined by an optical camera to reveal the surface morphology. Figure 5 shows the photos of polymeric composite films. As shown in Figure 5a, low dispersion of iron powder with severe agglomeration was observed for IPMMA-4. Although the iron powder exhibited a relatively uniform distribution in IPVDF-4 (Figure 5b), some clusters and agglomerations of iron particles were observed. In contrast, IPVA-4 (Figure 5c) exhibited a uniform film without obvious agglomeration and showed a homogenous filling of iron powder. The surface morphology of composite films was further examined by an optical microscope. Figure 6a showed the surface image of IPMMA-4 composite films that exhibited a rough surface with agglomeration of iron powder (the white part in Figure 6a) in the PMMA matrix. A similar result was observed for IPVDF-4, Figure 6b. However, the agglomeration was more severe than within PMMA. For the IPVA-4 (Figure 6c), the distribution of iron powder was more uniform than those in PMMA (Figure 4a) and PVDF (Figure 6b). This suggests that the dispersibility of iron powder in PVA was better than the other two composite films. Detailed surface morphology was examined further by SEM. With low magnification SEM images of IPMMA-4, Figure 7a, lots of small bright spots were observed on the rough surface, which resulted from the agglomeration of the iron powder in the PMMA film. Different from the IPMMA-4, no distinct bright spots can be observed in IPVDF-4 and IPVA-4 composite films, as shown in Figure 7b,c, respectively. The iron powder distribution in the polymeric composite films also can be observed in SEM images with high magnification, as shown in Figure 7d–f. The high magnification SEM image of IPMMA-4 (Figure 7d) revealed obvious agglomeration of iron powder. For the IPVDF-4 (Figure 7e), iron powders were hardly visible at low magnification but appeared as aggregated clusters under higher magnification images. In contrast, the IPVA-4 exhibited a significant uniform distribution of iron powder in Figure 7e. The photo (Figure 5), OM (Figure 6), and SEM (Figure 7) observation reveal that the dispersibility of iron powder within polymers was the best for IPVA-4 and the worst for IPVDF-4, with IPMMA-4 in between.

**Figure 8:**
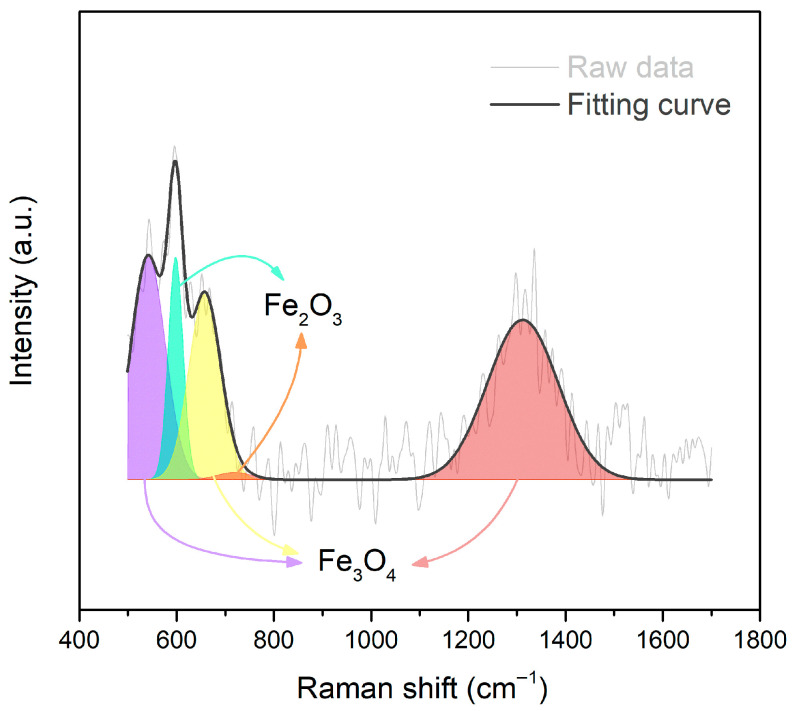
The Raman spectrum of iron powder with its fitting curve and deconvoluted areas for Fe_3_O_4_ (purple, yellow, and red areas) and Fe_2_O_3_ (cyan and orange areas).

Figure 8 shows the Raman spectrum of the iron powder that exhibited the characteristic peaks at 539, 598, 671, 719, and 1336 cm^−1^, respectively. The spectrum was further fitted, deconvoluted, and identified as Fe_3_O_4_ (at 539, 671, and 1336 cm^−1^) and the Fe_2_O_3_ (at 598 and 719 cm^−1^), respectively. Although no iron oxides were observed in the XRD pattern (Figure 2a), the Raman spectrum revealed the existence of iron oxides, including Fe_3_O_4_ and Fe_2_O_3_, in the iron powder. The XRD and Raman spectrum results suggest that iron powder possessed a thin oxide layer that can be easily attached to the -OH functional groups in the PVA. This may be attributed to the relatively uniform dispersion of the iron within the PVA matrix. Furthermore, PVA was dissolved by water solvent. The interaction between iron oxides, -OH, and water hydrogen bonding may also reduce the possibility of iron powder agglomeration in the PVA composite film.

### 3.2. Sheet Resistance Adjustment of Polymeric Composite Films

**Figure 9:**
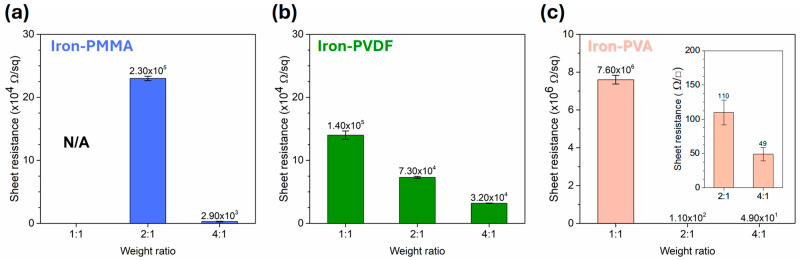
The sheet resistance of various iron powder added amounts of (**a**) PMMA, (**b**) PVDF, and (**c**) PVA composite films.

Three iron-added polymeric composite films, IPMMA, IPVDF, and IPVA, with various contents of iron powders were examined by a four-point probe to obtain the sheet resistance. Figure 9a shows the sheet resistance of IPMMA in the weight ratios of 1:1, 2:1, and 4:1. No sheet resistance value (out of scale) was available for IPMMA-1. With increasing iron ratio, the sheet resistance value was 2.30 × 10^5^ and 2.90 × 10^3^ Ω/sq for IPMMA-2 and -4, respectively. The sheet resistance decreased with increasing adding amount of iron powder. A similar trend was observed for IPVDF (Figure 9b) and IPVA (Figure 9c). The values of the sheet resistance were 1.40 × 10^5^, 7.30 × 10^4^, and 3.20 × 10^4^ Ω/sq for IPVDF-1, -2, and -4 composite films, respectively. For IPVA films, it should be pointed out that the sheet resistance value sharply decreased from 7.60 × 10^6^ (IPVA-1) to 1.10 × 10^2^ (IPVA-2) and 49.0 ± 9.7 Ω/sq (IPVA-4). Iron powder exhibited better dispersion within the PVA matrix than PMMA and PVDF, thereby lowering the sheet resistance values.

**Figure 10:**
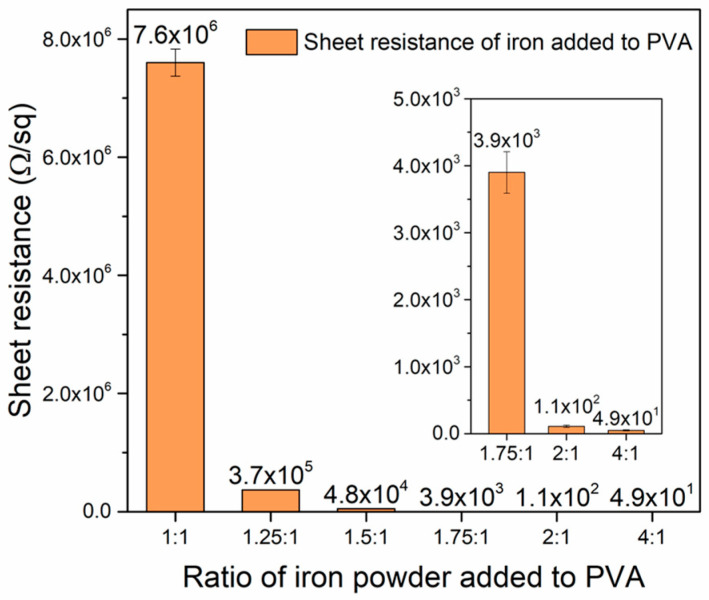
The sheet resistance of the IPVA composite films prepared with various iron-PVA ratios.

Figure 10 shows the sheet resistance of the IPVA with the refined iron content between 1:1 and 2:1. It can be noted that the sheet resistance rapidly decreased from 7.60 × 10^6^ (IPVA-1) to 3.7 × 10^5^ Ω/sq for IPVA-1.25. Following the increase in iron content, the sheet resistance exhibited a slow decrease. The sheet resistances of IPVA-1.5 and IPVA-1.75 were 4.8 × 10^4^ and 3.9 × 10^3^ Ω/sq, respectively. Although the sheet resistance of IPVA-1.25 to -1.75 decreased with the increasing iron content, the values were still too high for matching with air-impedance (~377 Ω). The IPVA-2 with a sheet resistance of 1.10 × 10^2^Ω/sq exhibited closer sheet resistance to air-impedance than IPVA-4 (49.0 ± 9.7 Ω/sq). The sheet resistance, however, is expected to increase after adding microwave efficient-absorption material. Thus, IPVA-4 was selected to add various contents of the Fe nanowire for impedance-matching examination.

**Figure 11:**
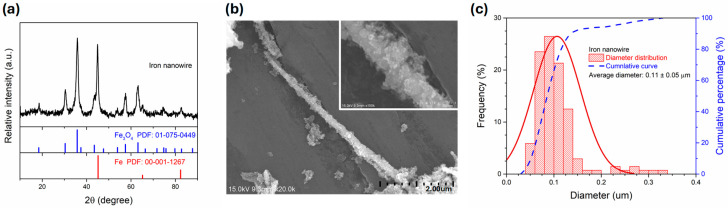
(**a**) XRD pattern, (**b**) SEM images, and (**c**) diameter distribution of as-synthesized Fe nanowires.

Figure 11 shows the XRD, SEM, and diameter distribution of the iron nanowire that will be used as the microwave absorbers within impedance-matched polymeric composite film. The observed characteristic peaks of the Fe nanowire (Figure 11a) indicate that both Fe (ICDD PDF No. 00-001-1267) and Fe_3_O_4_ (ICDD PDF No. 00-075-0449) phases existed. Figure 11b shows the SEM images of Fe nanowire. The rough surface of the Fe nanowire indicated that the Fe_3_O_4_ layer was on the outside surface of the Fe nanowire, as shown by the inset image in Figure 11b [55,67,68]. The diameter distribution of the Fe nanowire was further measured using image J and the result was shown in Figure 11c. The average diameter of Fe nanowire was 0.11 ± 0.05 μm.

**Figure 12:**
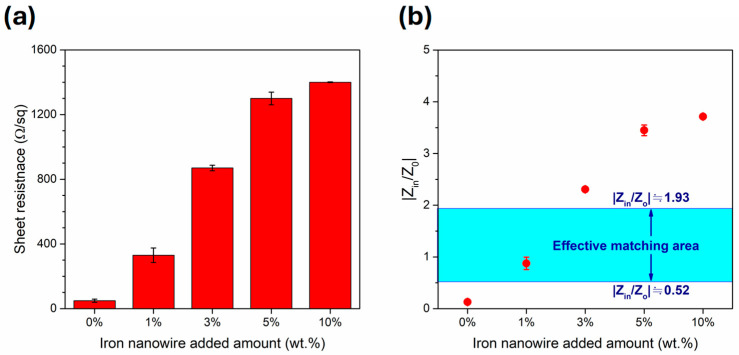
The (**a**) sheet resistance and (**b**) |Z_in_/Z_0_| values (red symbol) of IPVA-4 composite films with different amounts of Fe nanowire additions (0, 1, 3, 5, and 10 wt.%).

The as-prepared Fe nanowires (microwave absorbers) were applied to adjust the sheet resistance for impedance matching (~377 Ω). Figure 12a shows the sheet resistance of IPVA-4 with various Fe nanowire additions (0, 1, 3, 5, and 10 wt.%). It can be noted that the sheet resistances increased with increasing amount of iron nanowire additions, probably due to the outside Fe_3_O_4_ layer of Fe nanowire. It was 329.7 ± 45.3 Ω/sq for 1 wt.% addition and increased sharply to 866.8 ± 17.4, 1268.6 ± 38.6, and 1433.4 ± 12.1 Ω/sq for 3, 5, and 10 wt.%, respectively. Though the distribution of Fe nanowire may affect the sheet resistance, the standard deviation was probably attributed to the measurement uncertainty. In the Salisbury screen-type absorber, when the thickness of the resistance sheet is smaller than skin depth (δ), the sheet resistance can be indicated as the value of Z_in_ [69]. The δ value is defined as the thickness below the outer surface at which the incident field is attenuated to 1/*e* of its initial value [70], and can be calculated by δ = (*fπσμ*)^−1/2^, where *f* is the incident frequency, *μ* is the relative permeability, and *σ* is the conductivity of the resistance sheet. The *μ* values of the composites can be referred from our previous report showing that the iron powder and iron nanowire-added composite were approximately 1.00 [55]. The δ value of various Fe nanowire additions can be obtained at frequencies of 2, 10, and 18 GHz, and listed in Table 1. The obtained δ values were larger than the as-prepared composite thickness (100 μm) in the frequency range from 2 to 18 GHz. Thus, the effect of electromagnetic properties (permittivity and permeability) in the resistance sheet can be ignored. The impedance matching degree can be calculated by the |Z_in_/Z_0_| value where Z_in_ and Z_0_ is the impedance value of the film and the air, respectively. The calculated |Z_in_/Z_0_| values were 0.13 ± 0.03, 0.88 ± 0.12, 2.31 ± 0.05, 3.45 ± 0.10, and 3.71 ± 0.01 for 0, 1, 3, 5, and 10 wt.% Fe nanowire addition, respectively. These results were shown as the red dots in Figure 12b. Moreover, the efficient |Z_in_/Z_0_| value can be calculated from the RL(dB) = −20log(|Z_in_ − Z_0_|/|Z_in_ + Z_0_|), when RL ≤ −10 dB. The efficient |Z_in_/Z_0_| value fell within 0.52 to 1.93, as shown in the light blue area in Figure 12b. It should be noted that IPVA-4 with 1 wt.% Fe nanowire addition exhibited well impedance matching and was the best composition obtained in the present study. This behavior is mainly attributed to the microstructural and interfacial effects induced by the iron/Fe nanowire fillers, which modifies the overall sheet resistance within the polymer matrix, thereby improving the impedance matching performance.

Based on the above findings and analyses, several points regarding the current limitations and future perspectives of this study are worth noting. Although the present study determined the impedance-matching behavior of the iron-added polymeric composite films, the evaluation of absorption performance in the Salisbury screen-type absorber is necessary. Furthermore, the electromagnetic properties were not considered in this study; however, the spacer thickness still related to the dielectric constant of the resistance sheet and influenced the absorption performance. The future work for this study will therefore focus on the evaluation of the reflection loss efficiency in the Salisbury screen-type absorber by free-space measurement. The present results offer a practical guidance, serving as a reference for future applications in pattern design and multilayer resistance sheets for Salisbury screen-type absorbers.

## 4. Conclusions

In the present study, Salisbury screen-type impedance-matched resistance sheets were prepared by iron-added PMMA, PVDF, and PVA polymeric composite films with iron nanowire additions. The sheet resistance decreased with increasing iron addition within polymeric matrices. Due to the well-dispersed iron powder in the PVA polymer matrix, IPVA-4 (iron-PVA = 4:1) exhibited the lowest sheet resistance of 4.9 × 10^1^ Ω/sq. With 1 wt.% Fe nanowire addition into IPVA-4, the sheet resistance was 329.7 ± 45.3 Ω/sq, the |Z_in_/Z_0_| value reached 0.88 ± 0.12, and it exhibited good impedance matching. This iron-added polymeric composite film tuned by Fe nanowire addition provided a strategy for impedance matching and microwave absorption.

## Figures and Tables

**Table 1:** The δ values of various Fe nanowire additions in IPVA-4 at 2 GHz, 10 GHz, and 18 GHz.

Fe Nanowire Content	Sheet Resistance (Ω/sq)	σ (S/m)	δ_2 GHz_ (μm)	δ_10 GHz_ (μm)	δ_18 GHz_ (μm)
0 wt.%	49.0 ± 9.7	204.1 ± 59.5	787.8	352.3	262.6
1 wt.%	329.7 ± 45.3	30.3 ± 6.0	2043.5	913.9	681.2
3 wt.%	866.8 ± 17.4	11.5 ± 0.3	3313.3	1481.8	1104.4
5 wt.%	1268.6 ± 38.6	7.9 ± 0.3	4008.4	1792.6	1336.1
10 wt.%	1433.4 ± 12.1	7.0 ± 0.0	4260.8	1905.5	1420.3

## Data Availability

The original contributions presented in this study are included in the article. Further inquiries can be directed to the corresponding authors.
